# Making the case for the family model in in-patient child and adolescent mental healthcare

**DOI:** 10.1192/bjb.2021.8

**Published:** 2021-10

**Authors:** Lesley Cousins, Joanne Holmes

**Affiliations:** 1 Croft Child and Family Unit, Fulbourn, UK

**Keywords:** Carers, childhood experience, in-patient treatment, trauma, psychiatric nursing

## Abstract

Within paediatrics, young children experiencing physical and emotional distress are admitted to hospital with their parents as a matter of course, recognising the trauma associated with parting children from their carers. Much of this practice is underpinned by our understanding of attachment theory, which also sits as a fundamental tenet of child psychiatry. Yet the culture in psychiatric in-patient hospitals remains to admit young children without their parents, often to units that are geographically distant from the family home. We argue that the practice of admitting lone children to psychiatric in-patient units is likely to be traumatising as well as less effective. We believe this culture requires challenge and change.

There are currently only seven child and adolescent mental health (CAMH) in-patient units admitting children under the age of 13 in the UK. Of these, just one admits children along with their parent or carer as a matter of course.^1^ We believe this standard practice of admitting children without an accompanying parent/carer to be outdated and detrimental to both child and family.

Enter a paediatric ward today and the sight of a parent accompanying their sick child would be commonplace. Indeed, for children of primary school age, and especially those requiring extended treatment over weeks or months, if a parent or carer were not present for much of the admission there would be concerns raised about the adequacy of the care they were receiving from the family. Over the past 60 years, our paediatric colleagues have increasingly recognised the value of parents accompanying their child in order to minimise the child's distress and to improve overall outcomes.^2^ Yet we in CAMH in-patient services continue to admit lone children, often to facilities that are a considerable distance from the family home.

The initial recognition of the trauma caused to children separated from their parents by hospital admission, and the resulting movement to allow parents to stay alongside their children, was significantly led by John Bowlby.^3^ Bowlby's work in the development of attachment theory has been integral to our understanding of child emotional development and underlies much of our current practice of child psychiatry. Many research studies have confirmed the association between insecure relational family attachment patterns and childhood emotional and behavioural difficulties,^4^ and it is therefore not surprising that we see an increased prevalence of insecure attachment patterns in the clinical population of the children we care for.^5,6^ Given this, it seems illogical that in-patient CAMH practice continues to admit unaccompanied children, placing children at risk of the double trauma of separation from parents and admission to a mental health setting.

Children exist within and respond to the wider system around them, of which the family is the predominant part. Often the journey that brings a child to an in-patient admission involves the breakdown and disruption of this family system.^7^ Any therapeutic intervention must take this into account and address it. The attachment relationship between a parent and child is a dynamic process that we believe can only be really understood by directly observing their interaction. Admitting the parent and child together allows clinicians to assess attachment patterns directly and to establish to what extent disruption in relational security is driving and/or maintaining the child's mental disorder. It is not uncommon for a parent's own emotional difficulties to affect their attachment relationship and we know that a parent's mental health represents the most important correlate for all domains of the child's potential mental health difficulties.^8^ Many parents of children in in-patient CAMH services have significant backgrounds of trauma and it is common to see the effects of intergenerational trauma displayed in a child's behaviour or for a traumatic response to be triggered in the parent by this behaviour. This understandably can make it impossible for any parent to implement any behavioural strategies suggested by professionals. If parents are part of the admission, this helps the team to develop the trust required to undertake the therapeutic work needed and address these issues more readily.

We should not be surprised that some parents understandably find the idea of abandoning their distressed, frightened child with professionals in an institution far from home for a potentially prolonged period unacceptable. This may mean that the family refuse the admission that they need. By preventing parents from accompanying their children are we denying families the potential to get the help they require? Alternatively, some parents, exhausted by the challenge of trying to care for their child, may be eager to accept an admission to hospital, thus gaining respite from the responsibility of care. However, a hospital admission can strengthen the medicalisation of the child's difficulties, and handing over the care of the child to a clinical team may further promote a family's belief that the child needs to be ‘fixed’ by professionals or disempowers parents if the child's symptoms dissipate once they are in a hospital setting.

Even if a child's mental disorders are not driven by disrupted relationships, it is vital that the parents/carers can be fully involved in their child's care, as they will become the child's care team on discharge. In all cases the parents/carers of a young child need to become part of the solution for that child rather than continuing to fuel the problem or to believe that only professionals can help.

## Are we denying children the opportunity to get the help by not routinely admitting parents with them?

Although we strongly believe that the benefits of parents being admitted with their children to in-patient CAMH settings are clear and meaningful, we recognise that, given this is not routine, there are clearly arguments opposing it.

There is evidence that the practice of admitting lone children and focusing on individual work with the child alone is effective.^7,9^ It may therefore be argued that the additional admission of the parent is unnecessary. A study measuring the outcomes of child in-patient admissions in England and Wales demonstrated their effectiveness and calculated the average cost of a child in-patient admission, but it did not compare the units that do not admit parents with the one that does.^7^ We therefore do not know whether this assumption is correct and clear evidence exists that more can be achieved when children are admitted with their parent or carer than when the child is admitted alone.^10^

We note that some children with emotional or behavioural difficulties can benefit from time away from the family home, particularly when ongoing stress in the child–parent relationship is the main source of their distress. We would argue that removing the child from the home temporally is unlikely to address the difficulties long term and intensive work is required with parents to make the systemic change that will be required for the child to benefit when discharged. Although this family work can, and does, happen on an out-patient basis, it is often difficult for parents to attend regularly,^11^ particularly when children are admitted a long way from home.

Finally, we also recognise the considerable financial implications as well as disruption to family life for siblings at home when a parent is admitted with their child.^7^ Again, we would argue that the long-term consequences of having a child with significant behavioural and emotional problems is in itself disruptive and costly, with long-term negative consequences if not addressed. We would, however, also argue that the family's financial needs could be more actively addressed societally and appropriate reimbursement made available.

## Clinical implications and future plans

We have argued that a clinical model that promotes parents accompanying their child during an in-patient CAMH admission is beneficial, therapeutic and should be considered normal practice. We believe the current practice of admitting lone children under the age of 13 to be outdated and anti-therapeutic. We recognise that no specific clinical trials have been undertaken to support this model in mental health settings but we note that our paediatric colleagues have not been required to provide such evidence for their culture and the acceptance of parents on their wards to change; common sense has prevailed by itself. Admitting a child to an in-patient ward is a radical treatment option and including parents in this approach significantly adds to the intensity. We argue, however, that the involvement of parents or carers is fundamental to making the most of this period of family upheaval and is in the best interests of both the child and the family.

## About the authors

**Lesley Cousins**, MB, BChir, MRCPsych, DPhil, is a Year 6 Specialty Trainee (ST6) in child and adolescent mental health (CAMH), currently working at the Croft Child and Family Unit, Fulbourne, Cambridge, UK. **Joanne Holmes**, DM, MRCPsych, PG Cert MedEd, is a consultant CAMH psychiatrist based at the Croft Child and Family Unit, Fulbourne, Cambridge, UK.
